# Combined Prognostic Value of Preoperative Temporal Muscle Thickness and Geriatric Nutritional Risk Index in Surgically Treated Head and Neck Squamous Cell Carcinoma

**DOI:** 10.3390/cancers18142221

**Published:** 2026-07-10

**Authors:** Takuya Miura, Hisashi Kessoku, Yohei Morishita, Toshiki Kobayashi, Yosuke Mizunari, Shinichi Okada, Hiroto Ohto, Masato Nagaoka, Hiromi Kojima

**Affiliations:** 1 Department of Otorhinolaryngology and Head and Neck Surgery, Jikei University Kashiwa Hospital, Kashiwa 277-8567, Japan; takuyamiura0625@jikei.ac.jp (T.M.);; 2 Department of Otorhinolaryngology, Jikei University West Medical Center, Tokyo 201-8601, Japan; 3 Department of Otorhinolaryngology, Jikei University Hospital, Tokyo 105-0003, Japan

**Keywords:** temporal muscle thickness (TMT), geriatric nutritional risk index (GNRI), head and neck squamous cell carcinoma, prognosis, disease-free survival, overall survival, sarcopenia, nutritional status, risk assessment, computed tomography

## Abstract

**Background/Objectives**: Temporal muscle thickness (TMT) has been proposed as a practical surrogate marker for skeletal muscle mass, whereas the geriatric nutritional risk index (GNRI) reflects nutritional status. However, the independent and combined prognostic value of TMT and GNRI in surgically treated head and neck squamous cell carcinoma (HNSCC) remains unclear. **Methods**: We retrospectively analyzed 214 patients with HNSCC who underwent curative-intent surgery. Disease-free survival (DFS) and overall survival (OS) were evaluated using Cox proportional hazards models. In the primary analyses, TMT and GNRI were entered simultaneously as continuous variables and adjusted for age, sex, clinical stage, and postoperative adjuvant treatment. For clinical interpretability, Kaplan–Meier analyses were additionally performed using a composite TMT–GNRI score. **Results**: In the multivariable analyses, higher TMT was independently associated with longer DFS (hazard ratio [HR] 0.83 per 1 mm increase, 95% confidence interval [CI] 0.72–0.96, *p* = 0.014) and OS (HR 0.73, 95% CI 0.60–0.89, *p* = 0.002). GNRI was significantly associated with DFS and OS in the univariate analyses; after simultaneous adjustment for TMT and clinical covariates, it remained independently associated with OS (HR 0.98 per 1-point increase, 95% CI 0.96–0.99, *p* = 0.015) and showed a borderline association with DFS (HR 0.984, 95% CI 0.966–1.001, *p* = 0.068). Kaplan–Meier analyses using the composite TMT–GNRI score demonstrated clear risk stratification, with the poorest outcomes observed in patients with concomitantly low TMT and low GNRI. **Conclusions**: In surgically treated HNSCC, preoperative TMT was independently associated with both DFS and OS. GNRI may provide additional prognostic information, particularly for OS.

## Simple Summary

Outcomes after curative-intent surgery for head and neck squamous cell carcinoma vary considerably among patients. Simple preoperative markers of muscle quantity and nutritional status may help identify those at higher risk of recurrence and death. Temporal muscle thickness, measured on routine computed tomography, is a practical imaging-based surrogate of skeletal muscle quantity, whereas the geriatric nutritional risk index is a simple nutritional risk measure based on body weight and serum albumin. In this retrospective study, lower temporal muscle thickness was independently associated with shorter disease-free and overall survival. A lower geriatric nutritional risk index was additionally associated with shorter overall survival. Patients with both low temporal muscle thickness and a low geriatric nutritional risk index had the poorest outcomes. These findings suggest the potential utility of combining muscle and nutritional assessment before surgery for risk stratification in patients with head and neck cancer.

## 1. Introduction

Cancer prognosis is shaped by not only tumor burden but also the patient’s baseline physiological reserve. Loss of skeletal muscle mass has gained attention as a clinically meaningful component of body composition and has been associated with poor survival and greater treatment-related morbidity across multiple malignancies. In head and neck cancer, muscle depletion is common and has been linked to unfavorable survival outcomes and a higher risk of postoperative complications [1,2,3,4,5].

Sarcopenia is typically defined as low muscle mass accompanied by impaired muscle strength and reduced physical performance [6,7]. However, functional assessments are often unavailable in retrospective cohorts and routine practice, making imaging-derived measures useful as practical surrogate markers for muscle quantity. Cross-sectional muscle area at the third lumbar vertebra level is widely accepted as a reference measure, and third cervical vertebra-based muscle quantification has been adopted in head and neck cancer research [5,6,7]. However, third cervical vertebra muscle segmentation generally requires dedicated software and additional processing time, which can limit its routine applicability.

Temporal muscle thickness (TMT), measured using standard head computed tomography (CT) or magnetic resonance imaging (MRI), correlates with lumbar skeletal muscle metrics, including the psoas muscle [8], and has emerged as an accessible imaging marker of muscle depletion. TMT has also been proposed as a prognostic indicator in several malignancies [9,10,11]. In addition, nutritional status is a key determinant of outcomes in oncology. The geriatric nutritional risk index (GNRI), originally proposed for nutritional assessment in older inpatients [12], has been applied in diverse clinical settings, including cancer, and has been proposed as a prognostic marker in head and neck cancer [13,14,15,16,17,18,19,20].

TMT and GNRI capture different but interrelated domains—muscle quantity and nutritional risk—and may therefore provide complementary prognostic information. In several malignancies, including pancreatic cancer, intrahepatic cholangiocarcinoma, and diffuse large B-cell lymphoma, combining muscle-related indices with GNRI has improved prognostic stratification compared with either index alone [21,22,23]. However, the combined prognostic utility of TMT and GNRI remains to be directly validated in patients with head and neck cancer.

Accordingly, the present study aimed to evaluate the prognostic significance of preoperative TMT and GNRI in patients with head and neck squamous cell carcinoma (HNSCC) undergoing curative-intent primary surgery at our institution. Using multivariable Cox proportional hazards models, we assessed the independent associations of TMT and GNRI with disease-free survival (DFS) and overall survival (OS).

## 2. Materials and Methods

### 2.1. Study Design and Patients

This retrospective observational study included consecutive patients with histologically confirmed HNSCC who underwent curative-intent surgery at the Department of Otolaryngology–Head and Neck Surgery, Kashiwa Hospital, The Jikei University School of Medicine, between January 2015 and December 2023. Tumor staging was reassessed according to the 8th edition of the Union for International Cancer Control (UICC) TNM classification, including cases treated before 2018. The study was reported in accordance with the Strengthening the Reporting of Observational Studies in Epidemiology statement [24]. This study was approved by the Ethics Committee of The Jikei University School of Medicine and conducted in accordance with the Declaration of Helsinki (approval number: 37-465(13117); date of approval: 26 January 2026).

### 2.2. Inclusion and Exclusion Criteria

**Figure 1:**
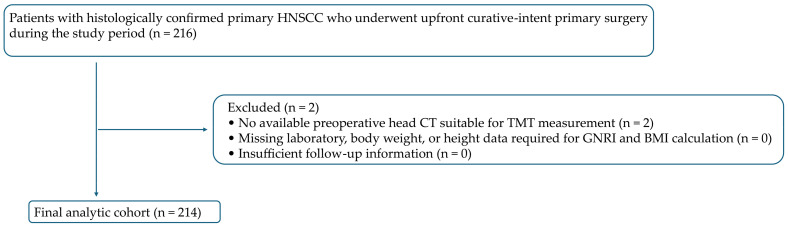
Flowchart of patient inclusion and exclusion. Abbreviations: BMI, body mass index; CT, computed tomography; GNRI, geriatric nutritional risk index; HNSCC, head and neck squamous cell carcinoma; TMT, temporal muscle thickness.

Patients were eligible if they underwent upfront curative-intent primary surgery for primary HNSCC at our institution, regardless of whether postoperative adjuvant therapy was administered. No additional clinical exclusion criteria were applied. For inclusion in the present analysis, patients were required to have routine preoperative clinical CT images that included adequate visualization for TMT measurement within six weeks before surgery, as well as preoperative laboratory data, including complete blood count and serum albumin, and body weight and height data required for calculation of the GNRI and body mass index (BMI), also obtained within six weeks before surgery. Patients who did not meet these analytic requirements were excluded from the final analysis. After application of these criteria, 214 patients constituted the final analytic cohort. The patient selection process is shown in Figure 1.

### 2.3. Data Collection

Baseline demographic data (age at surgery, sex, height, body weight, and BMI), tumor-related variables (primary site and clinical stage), treatment-related variables (reconstructive procedures and postoperative adjuvant treatment [none/radiotherapy/concurrent chemoradiotherapy]), and outcome variables were extracted from the electronic medical records. Preoperative blood tests, body measurements, and routine clinical CT images suitable for TMT measurement obtained within 6 weeks before surgery were used to calculate the study indices. In addition, selected pathological variables, including surgical margin status, extranodal extension, and perineural invasion, were extracted from the final pathological reports for additional sensitivity analyses.

### 2.4. CT Acquisition and TMT Measurement

TMT was measured on routine preoperative clinical CT images obtained as part of standard care, with a slice thickness of 3 mm. Both contrast-enhanced and non-contrast examinations were included, reflecting routine clinical practice. Images were reconstructed using a soft-tissue algorithm and evaluated with standard soft-tissue window settings.

**Figure 2:**
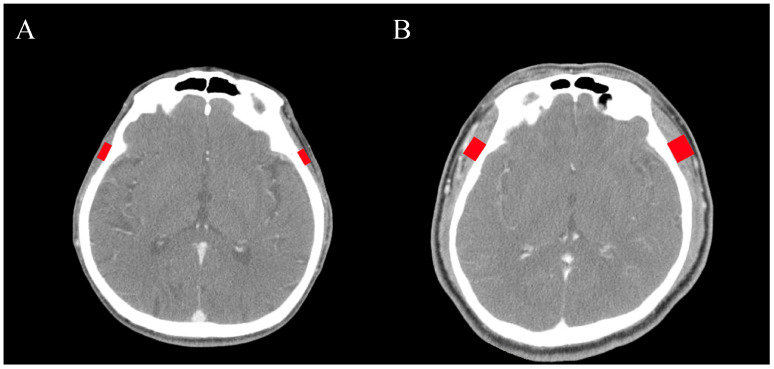
Measurement of TMT on axial computed tomography at the level of the superior orbital rim. (**A**) Representative axial CT image from a patient in the low TMT group. (**B**) Representative axial CT image from a patient in the high TMT group. Temporal muscle thickness was measured at the thickest portion of each temporal muscle, and the mean of the bilateral measurements was used for analysis. The red bars indicate the sites where temporal muscle thickness was measured. Abbreviations: CT, computed tomography; TMT, temporal muscle thickness.

The measurement plane was defined at the level of the superior orbital rim, and the slice on which both temporal muscles were most clearly visualized at this level was selected for measurement [25]. To reduce variability related to slice selection and head positioning, both observers used the same predefined anatomical landmarks and selected the slice at the superior orbital rim level on which the bilateral temporal muscles were most clearly visualized. For each side, the thickest portion of the temporal muscle belly was identified and measured as a linear distance perpendicular to the long axis of the muscle. The mean of the left and right measurements was used as the patient-level TMT value (Figure 2).

### 2.5. Interrater Measurement Procedure

Two observers independently measured TMT on preoperative axial CT images using the same anatomical landmarks and measurement protocol. Each observer measured the left and right TMT, and the mean of the two sides was used as the patient-level TMT value. When an interobserver discrepancy > 1.0 mm was identified, the images were jointly re-evaluated to confirm the landmarks and measurement plane. For the final analysis, the mean value of the patient-level TMT measurements performed by the two observers was used. Interrater reliability was assessed using the intraclass correlation coefficient (ICC) for continuous TMT values and Cohen’s kappa coefficient for dichotomized TMT status (low vs. high) based on predefined sex-specific cutoffs.

### 2.6. TMT Categorization

Sex-specific predefined cutoffs derived from a healthy young adult reference population were applied to dichotomize TMT [26]. Low TMT was defined as ≤6.3 mm in men and ≤5.2 mm in women, and patients were classified into either low or high TMT. These categorical definitions were used primarily for clinical interpretability and visualization, whereas TMT was analyzed as a continuous variable in the primary prognostic models.

### 2.7. GNRI Calculation

The GNRI was calculated as follows: GNRI = 1.489 × serum albumin (g/dL) + 41.7 × (actual body weight/ideal body weight) [12]. If the ratio of actual to ideal body weight exceeded 1.0, it was set to 1.0. Ideal body weight was defined assuming a body mass index of 22 kg/m^2^. Patient height data were obtained from the medical records and used to calculate ideal body weight. Based on prior studies, GNRI ≥ 98 (high GNRI) was defined as no nutrition-related risk, and GNRI < 98 (low GNRI) indicated risk of malnutrition [12]. In addition, GNRI was analyzed as a continuous variable (per 1-point increase) in Cox regression models to assess whether the observed associations were dependent on dichotomization (Supplementary Materials).

### 2.8. Definition of the TMT–GNRI Composite Score

A composite TMT–GNRI score was constructed to capture complementary domains of cancer-related vulnerability, namely skeletal muscle mass (TMT) and nutritional risk (GNRI). The score was defined a priori as a simple additive classification: patients with both favorable factors (high TMT and high GNRI) were assigned a score of 2, those with one unfavorable factor (high TMT/low GNRI or low TMT/high GNRI) were assigned a score of 1, and those with both unfavorable factors (low TMT and low GNRI) were assigned a score of 0. Patients were categorized into three groups (score 0–2) for survival analyses. Because the score 1 group combined two biologically distinct patterns, additional analyses using the four-category combination of TMT and GNRI were also performed as supplementary analyses. Alternative modeling approaches, including interaction terms, were also explored.

### 2.9. Outcomes and Follow-Up

DFS was defined as the time from the date of surgery to the first documented recurrence of HNSCC (local, regional, or distant) or death from any cause, whichever occurred first. OS was defined as the time from the date of surgery to death from any cause. Patients without an event were censored at the date of last clinical contact.

### 2.10. Statistical Analysis

Continuous variables were compared using the Mann–Whitney U test, whereas categorical variables were compared using the chi-square or Fisher’s exact test, as appropriate. Sex-specific cutoffs were used to dichotomize TMT (≤6.3 mm for men and ≤5.2 mm for women), and a GNRI cutoff of 98 was used to define low GNRI. Correlations among TMT, GNRI, and BMI were assessed using Pearson’s correlation coefficients. For the primary prognostic analyses, TMT and GNRI were modeled as continuous variables and entered simultaneously into multivariable Cox proportional hazards models to estimate their independent associations with survival outcomes. The primary multivariable model adjusted for age, sex, clinical stage (III–IV vs. I–II), and postoperative adjuvant treatment (yes/no). Because only 52 patients received postoperative adjuvant therapy, adjuvant treatment was coded as a binary covariate (yes/no) in the primary model to preserve model parsimony relative to the number of events. For sensitivity analyses, postoperative adjuvant treatment was modeled as a three-level variable (none, radiotherapy, and concurrent chemoradiotherapy), and the models were further adjusted for BMI, primary tumor site, and reconstructive surgery. Pathological variables were not included in the primary model because the primary objective of this study was to evaluate a preoperative prognostic model. However, in additional complete-case sensitivity analyses, we further adjusted the multivariable Cox models for surgical margin status, extranodal extension, and perineural invasion in patients with complete data for these pathological variables. Surgical margin status was analyzed as a binary variable (clear vs. non-clear), with close and positive margins included in the non-clear category. Extranodal extension was analyzed as absent vs. present, and perineural invasion was analyzed as absent vs. present.

For clinical interpretability and visualization, Kaplan–Meier curves were additionally presented using the categorical composite TMT–GNRI score (0–2). Supplementary analyses of the four-category combination and interaction terms were performed. Comparisons of baseline characteristics between groups (Table 1) were conducted for descriptive and exploratory purposes; therefore, p values were not adjusted for multiple testing and should be interpreted cautiously. DFS and OS were estimated using the Kaplan–Meier method and compared using the log-rank test. Univariate Cox proportional hazards analyses were performed as exploratory analyses.

The proportional hazards assumption was assessed using Schoenfeld residuals and global tests. Model discrimination and fit were evaluated using Harrell’s concordance index (C-index), Akaike Information Criterion (AIC), and Bayesian Information Criterion (BIC). Sensitivity analyses included alternative TMT definitions and continuous GNRI modeling, as well as pathology-adjusted complete-case models (Supplementary Materials). To evaluate the incremental prognostic value of combining TMT and GNRI, nested Cox models were compared using likelihood-ratio tests and information criteria (AIC and BIC). Differences in discrimination between models were assessed using bootstrap resampling (1000 replications) to estimate the difference in Harrell’s C-index (DeltaC). All analyses were performed using Stata/SE version 19.5 (StataCorp LLC., College Station, TX, USA), with a two-sided *p* value < 0.05 considered statistically significant.

## 3. Results

### 3.1. Patient Characteristics

The analytic cohort comprised 214 patients (Table 1). The mean age was 67.6 years (range, 39–89 years), and 184 patients (86.0%) were male. Primary tumor sites were oral cavity (*n* = 70, 32.7%), oropharynx (*n* = 52, 24.3%), hypopharynx (*n* = 56, 26.2%), and larynx (*n* = 36, 16.8%). Clinical staging according to the UICC 8th edition revealed stage I in 33 patients (15.4%), stage II in 30 (14.0%), stage III in 52 (24.3%), and stage IV in 99 patients (46.3%), constituting 63 patients (29.4%) in stage I–II and 151 (70.6%) in stage III–IV.

The mean TMT was 5.90 ± 1.75 mm (range, 2.61–12.8). Using sex-specific predefined cutoffs (≤6.3 mm for men and ≤5.2 mm for women), low TMT was observed in 117 patients (54.7%) and high TMT in 97 (45.3%). The mean BMI was 22.8 ± 3.6 (range, 12.1–37.3). The mean GNRI was 102.4 ± 12.5 (range, 44.7–133.2); low GNRI (<98) was present in 63 patients (29.4%) and high GNRI (≥98) in 151 (70.6%). Reconstructive skin flap surgery was performed in 149 patients (69.6%). Regarding postoperative adjuvant treatment, 162 patients (75.7%) received none, 28 (13.1%) received radiotherapy alone, and 24 (11.2%) received postoperative concurrent chemoradiotherapy.

### 3.2. Correlations Among TMT, GNRI, and BMI

TMT and GNRI showed a modest but significant positive correlation (Pearson r = 0.236, *p* = 0.0005; *n* = 214). BMI was weakly correlated with TMT (r = 0.209) and more strongly correlated with GNRI (r = 0.693). In a secondary analysis using dichotomized variables, low TMT was more frequent among patients with low GNRI (odds ratio 2.74, 95% confidence interval [CI] 1.46–5.17; chi-square *p* = 0.0015).

### 3.3. Survival Outcomes

The median follow-up period was 63.8 months (95% CI: 57.1–68.8 months). To assess the potential risk of informative censoring, we examined the proportion of censored patients with less than 24 months of follow-up; this accounted for 25 of 134 censored patients (18.7%) in the DFS analysis and 28 of 156 censored patients (17.9%) in the OS analysis. During follow-up, 80 DFS events and 58 deaths were observed. The 5-year DFS and OS rates were 63.1% (95% CI: 54.7–70.3%) and 74.2% (95% CI: 66.1–80.7%), respectively.

### 3.4. Prognostic Factors for DFS

In the univariate Cox proportional hazards analyses, lower TMT (hazard ratio [HR] 0.790 per 1 mm increase, 95% CI 0.686–0.909; *p* = 0.001), lower GNRI (HR 0.975 per 1-point increase, 95% CI 0.961–0.990; *p* = 0.001), and advanced clinical stage (III–IV vs. I–II) (HR 4.013, 95% CI 2.005–8.031; *p* < 0.001) were associated with worse DFS (Table 2). In the primary multivariable Cox model, in which TMT and GNRI were entered simultaneously as continuous variables and adjusted for age, sex, clinical stage (III–IV vs. I–II), and postoperative adjuvant treatment (yes/no), higher TMT remained independently associated with longer DFS (HR 0.833 per 1 mm increase, 95% CI 0.719–0.964; *p* = 0.014), whereas GNRI showed a borderline association (HR 0.984 per 1-point increase, 95% CI 0.966–1.001; *p* = 0.068). Advanced clinical stage remained a strong adverse prognostic factor (HR 3.851, 95% CI 1.869–7.938; *p* < 0.001). The proportional hazards assumption was not violated (global Schoenfeld test *p* = 0.789).

In a sensitivity analysis modeling postoperative adjuvant treatment as a three-level variable (none, RT, and CCRT), higher TMT remained independently associated with longer DFS (HR 0.835, 95% CI 0.721–0.967; *p* = 0.016), whereas GNRI was not significantly associated with DFS (HR 0.986, 95% CI 0.968–1.004; *p* = 0.130). The three-level postoperative adjuvant treatment variable was not significantly associated with DFS overall (overall *p* = 0.170).

In another sensitivity analysis incorporating p16 status as a three-level variable, higher TMT remained independently associated with DFS (HR 0.854, 95% CI 0.736–0.990; *p* = 0.037), whereas GNRI showed a borderline association (HR 0.984, 95% CI 0.966–1.003; *p* = 0.090). The overall p16 variable was not significantly associated with DFS (overall *p* = 0.168).

In additional sensitivity analyses, further adjusted for BMI, higher TMT remained independently associated with longer DFS, whereas GNRI was no longer significantly associated with DFS, and BMI itself was not independently associated with DFS. Additional sensitivity analyses further adjusted for primary tumor site and reconstructive surgery yielded results broadly consistent with the main findings, and TMT remained independently associated with DFS across all of these models (Supplementary Tables S1 and S2).

### 3.5. Prognostic Factors for OS

In the univariate analyses for OS, lower TMT (HR 0.662 per 1 mm increase, 95% CI 0.553–0.793; *p* < 0.001), lower GNRI (HR 0.965 per 1-point increase, 95% CI 0.949–0.981; *p* < 0.001), older age (HR 1.052 per year, 95% CI 1.023–1.082; *p* < 0.001), and advanced clinical stage (III–IV vs. I–II) (HR 6.435, 95% CI 2.330–17.774; *p* < 0.001) were associated with worse OS (Table 3).

In the primary multivariable model adjusted for age, sex, clinical stage (III–IV vs. I–II), and postoperative adjuvant treatment (yes/no), both higher TMT (HR 0.732 per 1 mm increase, 95% CI 0.604–0.888; *p* = 0.002) and higher GNRI (HR 0.975 per 1-point increase, 95% CI 0.955–0.995; *p* = 0.015) were independently associated with improved OS. Advanced clinical stage remained independently associated with worse OS (HR 5.114, 95% CI 1.804–14.495; *p* = 0.002; Table 3). The proportional hazards assumption was not violated (global Schoenfeld test *p* = 0.876).

In a sensitivity analysis modeling postoperative adjuvant treatment as a three-level variable (none, RT, and CCRT), higher TMT remained independently associated with longer OS (HR 0.727, 95% CI 0.600–0.880; *p* = 0.001), whereas the association of GNRI with OS was attenuated to borderline significance (HR 0.980, 95% CI 0.959–1.001; *p* = 0.065). The three-level postoperative adjuvant treatment variable was significantly associated with OS overall (overall *p* = 0.028).

In another sensitivity analysis incorporating p16 status as a three-level variable, higher TMT remained independently associated with OS (HR 0.760, 95% CI 0.625–0.924; *p* = 0.006), and higher GNRI also remained independently associated with OS (HR 0.975, 95% CI 0.953–0.996; *p* = 0.021). The overall p16 variable was not significantly associated with OS (overall *p* = 0.234).

In additional sensitivity analyses further adjusted for BMI, higher TMT remained independently associated with longer OS, whereas GNRI showed an attenuated and no longer statistically significant association with OS, and BMI itself was not independently associated with OS. Additional sensitivity analyses further adjusted for primary tumor site and reconstructive surgery yielded results broadly consistent with the main findings, with TMT remaining independently associated with OS across all of these models (Supplementary Tables S3 and S4).

### 3.6. Kaplan–Meier Analysis Using the Composite TMT–GNRI Score

**Figure 3:**
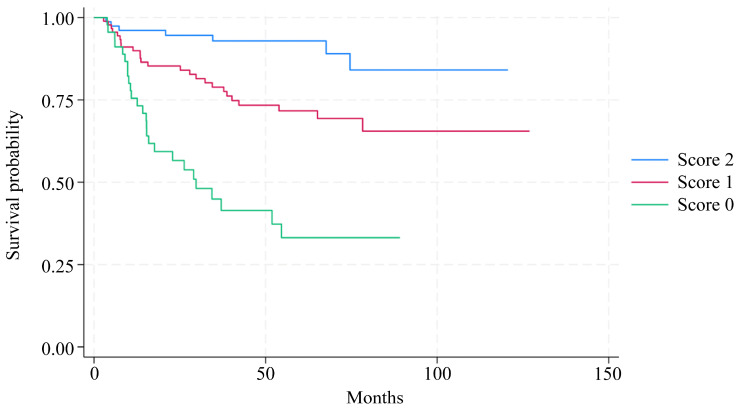
Kaplan–Meier curves for overall survival (OS) according to the composite TMT–GNRI score. Patients were classified into three groups (score 0–2), where score 0 indicated both unfavorable factors (low TMT and low GNRI), score 1 indicated one favorable factor, and score 2 indicated both favorable factors (high TMT and high GNRI). This composite score was used for visualization and clinical interpretability; primary inference was based on multivariable Cox models with TMT and GNRI entered as continuous variables. Survival differed significantly across groups (log-rank *p* < 0.001). Abbreviations: GNRI, geriatric nutritional risk index; TMT, temporal muscle thickness.

For visualization and clinical interpretability, Kaplan–Meier curves were generated using the three-group composite TMT–GNRI score (0–2). These curves demonstrated clear separation in OS across the combined TMT–GNRI groups, with the score 0 group exhibiting the lowest survival rates and the score 2 group showing the highest survival (Figure 3). Because score 1 merged two biologically distinct subgroups (high TMT/low GNRI and low TMT/high GNRI), additional Kaplan–Meier analyses using the four-category combination (high/low TMT × high/low GNRI) were performed (Figure S1). In these supplementary analyses, the two score 1 subgroups did not differ significantly in either OS (log-rank *p* = 0.512) or DFS (log-rank *p* = 0.909), supporting the use of the three-group composite score for simplified clinical visualization.

### 3.7. Interrater Reliability of TMT Measurement

Interrater reliability for TMT measurement was excellent (ICC: 0.988; 95% CI: 0.985–0.991). Agreement for dichotomized classification (low vs. high TMT) was also high (κ = 0.934; 95% CI: 0.784–1.000; Table 4).

### 3.8. Sensitivity Analyses

Sensitivity analyses using an alternative cohort-derived TMT categorization (sex-specific median split) yielded results broadly consistent with the main findings (Table S5). Additional analyses using a single-marker Cox model in which GNRI was entered as a continuous variable supported the robustness of the OS findings (Table S6). In further pathology-adjusted complete-case sensitivity analyses restricted to 189 patients with complete data for surgical margin status, extranodal extension, and perineural invasion, higher TMT remained independently associated with both DFS (HR 0.86, 95% CI 0.74–1.00, *p* = 0.044) and OS (HR 0.76, 95% CI 0.63–0.91, *p* = 0.003). GNRI was not significantly associated with DFS (HR 1.00, 95% CI 0.97–1.02, *p* = 0.701) but remained independently associated with OS (HR 0.98, 95% CI 0.96–1.00, *p* = 0.034). Extranodal extension and perineural invasion were independently associated with worse DFS and OS, whereas non-clear surgical margin status was not significantly associated with either outcome (Supplementary Table S7). Model discrimination analyses are presented in Table S8. Model comparison results of the incremental prognostic value of combining TMT and GNRI are presented in Table S9.

## 4. Discussion

In this retrospective study of patients with HNSCC treated with curative-intent surgery, we evaluated TMT and GNRI as prognostic markers. In the univariate analyses, both lower TMT and lower GNRI were associated with worse DFS and OS. In the primary multivariable Cox models in which TMT and GNRI were entered simultaneously as continuous variables and adjusted for age, sex, clinical stage, and postoperative adjuvant treatment, higher TMT remained independently associated with longer DFS and OS. GNRI showed a borderline independent association with DFS and remained independently associated with OS. These findings indicate that CT-derived TMT provides robust prognostic information for HNSCC beyond standard clinical factors, while GNRI may provide additional complementary prognostic information, particularly for OS, when evaluated concurrently. In additional pathology-adjusted complete-case sensitivity analyses, higher TMT remained independently associated with both DFS and OS, further supporting the robustness of the main findings. Although these analyses were restricted to patients with complete pathology data, extranodal extension and perineural invasion were also independently associated with worse outcomes, whereas surgical margin status was not significantly associated with either endpoint.

TMT has attracted attention as an imaging-based surrogate marker of skeletal muscle quantity. In 2017, Furtner et al. first reported that TMT measured on MRI is an independent prognostic factor in patients with brain metastases [26]. Subsequent studies demonstrated prognostic associations in glioblastoma [27] and brain metastases from malignant melanoma and lung cancer [28,29]. TMT also correlates with established measures of muscle mass and strength, including calf circumference [30], skeletal muscle mass at the third lumbar vertebra level [8], and muscle strength (including grip strength) [31], supporting its utility as a practical surrogate marker for sarcopenia [8,25,31,32]. In head and neck cancer, TMT has been linked to progression-free survival [33], but prior evidence has been limited by small cohorts and/or restricted primary sites, and OS has not been fully examined. Mukai et al. reported an association between low preoperative TMT and OS in oral squamous cell carcinoma [34]. Our study extends these observations by supporting the prognostic relevance of TMT across a broader surgical HNSCC population with diverse primary sites. Notably, because we did not directly assess muscle strength or physical performance, our results should not be interpreted as a comprehensive diagnosis of sarcopenia.

GNRI is a widely used nutritional index and has been reported as a prognostic marker in head and neck cancer [35,36]. In the present study, the added value of GNRI was not to replace TMT, but rather to complement it by capturing a related yet distinct domain of cancer-related vulnerability, including nutritional risk, systemic inflammation, and frailty. Accordingly, the principal novelty of our study lies in the combined evaluation of TMT and GNRI in surgically treated HNSCC. In our cohort, GNRI was significantly associated with DFS and OS in the univariate analyses. In the multivariable models that included TMT, the independent association of GNRI was weaker for DFS but remained evident for OS. One plausible interpretation is that TMT and GNRI capture partially overlapping domains of cancer-related vulnerability, whereas muscle quantity and nutritional risk may contribute differently to recurrence- and mortality-related outcomes. Consistently, we observed a modest positive correlation between TMT and GNRI, suggesting related but non-identical markers [37]. To further characterize the incremental value of combining these markers, we evaluated model discrimination metrics (Table S8) and compared nested Cox models to assess the added value of combining TMT and GNRI (Table S9). For OS, adding the second marker significantly improved model fit compared with either single-marker model, and the combined model showed the lowest AIC. For DFS, adding TMT to the GNRI-based model improved model fit, whereas adding GNRI to the TMT-based model did not yield a comparable improvement. Improvements in discrimination quantified by bootstrap differences in Harrell’s C-index were modest and did not consistently reach statistical significance. Model fit and discrimination capture different aspects of prognostic performance; thus, a significant improvement in fit does not necessarily translate into a large gain in discrimination when the added marker does not substantially change individual risk rankings, especially with limited event counts. However, because the association of GNRI with OS was attenuated to borderline significance in sensitivity analyses using alternative coding of postoperative adjuvant treatment, its incremental prognostic value should be interpreted as complementary rather than definitive.

For clinical interpretability and visualization, we performed Kaplan–Meier analyses using a categorical composite TMT–GNRI score (0–2). These curves showed clear risk separation for OS, with the poorest prognosis among patients with concomitantly low TMT and low GNRI. We positioned these categorical analyses as complementary rather than the primary basis for inference, because categorization of continuous variables can lead to information loss and reduced statistical power. Nevertheless, the composite score may be useful for communicating preoperative risk and for identifying a higher-risk subgroup that could be evaluated in future prospective interventional studies. Similar combined approaches integrating body composition indices with GNRI have improved risk stratification in other malignancies, including pancreatic cancer, intrahepatic cholangiocarcinoma, and diffuse large B-cell lymphoma [21,22,23]. Additional combination models and their results, including the four-category high/low TMT × high/low GNRI analysis, are provided in the Supplementary Materials.

Several biological mechanisms may explain the association between low TMT and adverse outcomes. Low TMT reflects reduced skeletal muscle mass and may coexist with impaired muscle quality, including intramuscular fat accumulation [38]. Such deterioration has been linked to metabolic dysregulation, as increased intramuscular triglycerides may exacerbate systemic insulin resistance and chronic inflammation [39], potentially promoting tumor progression and worse outcomes. Moreover, decreased serum albumin, a component of GNRI, may indicate impaired patient’s condition through susceptibility to infection and oxidative stress [40,41], contributing to immunosuppression within the tumor microenvironment and facilitating tumor progression [42]. Systemic metabolic alterations, including weight loss and worsening nutritional status, may also impair antitumor T-cell function and host immunity [43]. Together, these pathways may contribute to the poor prognosis observed among patients with depleted muscle reserves and nutritional risk.

From a clinical perspective, sarcopenia is associated with diminished physical function and an increased risk of long-term mortality [44,45,46,47,48,49,50]. Exercise and nutritional interventions can prevent or improve sarcopenia within certain settings [44,46]. In the context of head and neck cancer, early nutritional support reportedly improves outcomes [51]. Given that TMT can be measured using routinely acquired preoperative clinical CT images, it may serve as a practical marker for preoperative risk stratification. However, the present retrospective observational study does not demonstrate the efficacy of any specific nutritional or rehabilitative intervention. Rather, our findings suggest that patients with low TMT, particularly when accompanied by low GNRI, may represent a higher-risk subgroup that could be evaluated in future prospective interventional studies and may warrant closer postoperative surveillance.

This study has several limitations. First, its retrospective single-center design and the predominance of male Japanese patients in the cohort may limit the generalizability of the findings and may also have introduced selection bias and residual confounding. Second, although sensitivity analyses incorporating p16 status did not materially alter the main findings, residual confounding related to HPV/p16 status cannot be fully excluded, particularly in the oropharyngeal cancer subset. Third, because this study was based on routine preoperative clinical CT images rather than a protocol dedicated to TMT assessment, imaging acquisition parameters, head position, and scan coverage were not fully standardized. Although predefined anatomical landmarks were used and only scans with adequate visualization for TMT measurement were included in the analytic cohort, residual measurement error related to slice selection, acquisition variability, and scan coverage cannot be excluded. Fourth, while TMT offers an objective, imaging-based evaluation of muscle quantity, functional metrics such as grip strength and physical performance were not assessed, despite their importance as integral components of current sarcopenia definitions. Fifth, intra-rater reliability was not formally assessed because each case was measured once by each observer according to the predefined protocol. Sixth, although categorical classifications and composite scores may be clinically useful for visualization and risk communication, they can reduce informational content and statistical power; therefore, these categorical findings should be interpreted as complementary rather than primary. Nevertheless, sensitivity analyses employing alternative TMT definitions and modeling approaches supported the robustness of the primary conclusions.

## 5. Conclusions

In summary, preoperative TMT was independently associated with DFS and OS among patients undergoing surgical treatment for HNSCC, suggesting its potential value as a practical marker for preoperative risk stratification. GNRI may provide additional complementary prognostic information, particularly for OS. Together, these findings support the concept that concurrent evaluation of skeletal muscle mass and nutritional status may improve risk stratification in this patient group. Further prospective validation in larger and more diverse cohorts is warranted.

## Figures and Tables

**Table 1:** Baseline patient characteristics (*n* = 214).

Characteristic	Value
Age, years	
Mean ± SD (range)	67.6 ± 10.8 (39–89)
Sex, *n* (%)	
Male	184 (86.0)
Female	30 (14.0)
Clinical stage, *n* (%)	
I	33 (15.4)
II	30 (14.0)
III	52 (24.3)
IV	99 (46.3)
Stage grouping, *n* (%)	
I–II	63 (29.4)
III–IV	151 (70.6)
Primary tumor site, *n* (%)	
Oral cavity	70 (32.7)
Oropharynx	52 (24.3)
Hypopharynx	56 (26.2)
Larynx	36 (16.8)
Reconstructive surgery, *n* (%)	
Yes	149 (69.6)
No	65 (30.4)
Postoperative adjuvant treatment, *n* (%)	
None	162 (75.7)
Radiotherapy (RT)	28 (13.1)
Concurrent chemoradiotherapy (CCRT)	24 (11.2)
TMT, mm, mean ± SD	5.90 ± 1.75
Low TMT, *n* (%)	117 (54.7)
High TMT, *n* (%)	97 (45.3)
BMI, mean ± SD (range)	22.8 ± 3.6 (12.1–37.3)
GNRI, mean ± SD	102.4 ± 12.5
Low GNRI (<98), *n* (%)	63 (29.4)
High GNRI (≥98), *n* (%)	151 (70.6)

Values are presented as *n* (%) or mean ± SD unless otherwise indicated. Abbreviations: BMI, body mass index; TMT, temporal muscle thickness; GNRI, geriatric nutritional risk index; RT, radiotherapy; CCRT, concurrent chemoradiotherapy; SD, standard deviation.

**Table 2:** Univariate and multivariate analyses of the factors influencing disease-free survival.

Variable	Univariate Analysis		Multivariate Analysis	
	Hazard Ratio (95% CI)	*p* Value	Hazard Ratio (95% CI)	*p* Value
Age	1.02 (1.00–1.04)	0.074	0.997 (0.973–1.023)	0.828
Sex				
Female	Reference	-	Reference	
Male	1.139 (0.616–2.104)	0.678	1.331 (0.709–2.500)	0.374
Clinical stage				
I–II	Reference	-	Reference	
III–IV	4.013 (2.005–8.031)	<0.001 *	3.851 (1.869–7.938)	<0.001 *
Postoperative adjuvant treatment				
Yes	Reference	-	Reference	
No	0.832 (0.401–1.728)	0.622	0.578 (0.270–1.238)	0.158
TMT				
(per 1 mm increase)	0.790 (0.686–0.909)	0.001 *	0.833 (0.719–0.964)	0.014 *
GNRI				
(per 1-point increase)	0.975 (0.961–0.990)	0.001 *	0.984 (0.966–1.001)	0.068

In the multivariable Cox proportional hazards model, TMT and GNRI were entered simultaneously as continuous variables and adjusted for age, sex, clinical stage, and postoperative adjuvant treatment (yes/no). * Statistical significance (*p* < 0.05). Abbreviations: CI, confidence interval; GNRI, geriatric nutritional risk index; TMT, temporal muscle thickness.

**Table 3:** Univariate and multivariate analyses of the factors influencing overall survival.

Variable	Univariate Analysis		Multivariate Analysis	
	Hazard Ratio (95% CI)	*p* Value	Hazard Ratio (95% CI)	*p* Value
Age	1.052 (1.023–1.082)	<0.001 *	1.028 (0.995–1.062)	0.101
Sex				
Female	Reference	-	Reference	
Male	0.923 (0.419–2.035)	0.843	1.046 (0.465–2.352)	0.913
Clinical stage				
I–II	Reference	-	Reference	
III–IV	6.435 (2.330–17.774)	<0.001 *	5.114 (1.804–14.495)	0.002 *
Postoperative adjuvant treatment				
Yes	Reference	-	Reference	
No	0.594 (0.215–1.641)	0.315	0.839 (0.460–1.531)	0.568
TMT				
(per 1 mm increase)	0.662 (0.553–0.793)	<0.001 *	0.732 (0.604–0.888)	0.002 *
GNRI				
(per 1-point increase)	0.965 (0.949–0.981)	<0.001 *	0.975 (0.955–0.995)	0.015 *

The multivariable Cox proportional hazards model included age, sex, clinical stage, and postoperative adjuvant treatment (yes/no), with TMT and GNRI entered simultaneously as continuous variables. * Statistical significance (*p* < 0.05). Abbreviations: CI, confidence interval; GNRI, geriatric nutritional risk index; TMT, temporal muscle thickness.

**Table 4:** Interrater reliability of temporal muscle thickness (TMT) measurements (*n* = 214).

		Evaluator 2		
		Low TMT	High TMT	Total
Evaluator 1	Low TMT	116	1	117
	High TMT	6	91	97

Interrater reliability was assessed using the intraclass correlation coefficient (ICC) for continuous TMT values and Cohen’s kappa (κ) for dichotomized TMT classification (low vs. high). The ICC was 0.988 (95% CI, 0.985–0.991), and the κ was 0.934. Abbreviation: CI, confidence interval; TMT, temporal muscle thickness.

## Data Availability

The data supporting the findings of this study are available from the corresponding author upon reasonable request. The data are not publicly available due to institutional privacy restrictions.

## Supplementary Materials

The following supporting information can be downloaded at: https://www.mdpi.com/article/10.3390/cancers18142221/s1, Figure S1: Kaplan–Meier curves for DFS and OS across the four-category combination of TMT and GNRI; Table S1: Sensitivity analysis for disease-free survival additionally adjusted for primary tumor site; Table S2: Sensitivity analysis for disease-free survival additionally adjusted for reconstructive surgery; Table S3: Sensitivity analysis for overall survival additionally adjusted for primary tumor site; Table S4: Sensitivity analysis for overall survival additionally adjusted for reconstructive surgery; Table S5: Sensitivity analysis using cohort-derived sex-specific median cutoffs for TMT; Table S6: Single-marker Cox model for overall survival with GNRI entered as a continuous variable; Table S7: Pathology-adjusted complete-case sensitivity analyses for disease-free survival and overall survival; Table S8: Model discrimination for overall survival assessed by optimism-corrected concordance index (C-index); Table S9: Model comparison and incremental value of adding TMT and GNRI for DFS and OS.
