# Supplementary material for: Combined Prognostic Value of Preoperative Temporal Muscle Thickness and Geriatric Nutritional Risk Index in Surgically Treated Head and Neck Squamous Cell Carcinoma
Source: Cancers (Basel). 2026 Jul 10;18(14):2221. doi: 10.3390/cancers18142221 (PMC13407150; doi:10.3390/cancers18142221)
Supplement: Supplementary file 1 [file cancers-18-02221-s001.zip › cancers-4259609-supplementary.pdf]

Supplementary Materials

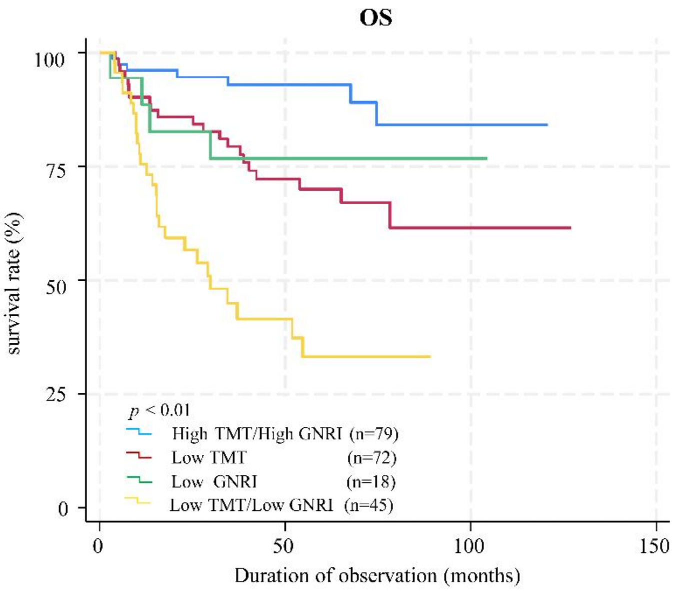

Figure S1. Kaplan–Meier curves for DFS and OS across the four-category combination of TMT and GNRI.

Table S1. Sensitivity analysis for disease-free survival additionally adjusted for primary tumor site.

**Variable**	**Multivariate analysis**	
	**Hazard ratio (95% CI)**	***p* value**
**AGE**	1.006 (0.979–1.033)	0.677
**SEX**		
Female	Reference	–
Male	0.748 (0.371–1.508)	0.417
**CLINICAL STAGE**		
I–II	Reference	–
III–IV	3.331 (1.531–7.246)	0.002*
**POSTOPERATIVE ADJUVANT TREATMENT**		
Yes	Reference	–
No	0.947 (0.527–1.701)	0.855
**TMT**		
(per 1-mm increase)	0.842 (0.728–0.974)	0.021*
**GNRI**		
(per 1-point increase)	0.983 (0.964–1.002)	0.077
**PRIMARY TUMOR SITE**		
Oral cavity	Reference	–
Oropharynx	0.455 (0.223–0.930)	0.031*
Hypopharynx	0.847 (0.472–1.519)	0.577
Larynx	0.672 (0.340–1.330)	0.254

Abbreviations: CI, confidence interval; GNRI, geriatric nutritional risk index; TMT, temporal muscle thickness. * Statistical significance ($p < 0.05$).

Table S2. Sensitivity analysis for disease-free survival additionally adjusted for reconstructive surgery.

**Variable**	**Multivariate analysis**	
	**Hazard ratio (95% CI)**	***p* value**
**AGE**	1.012 (0.983–1.042)	0.419
**SEX**		
Female	Reference	–
Male	0.883 (0.407–1.920)	0.754
**CLINICAL STAGE**		
I–II	Reference	–
III–IV	3.304 (1.539–7.094)	0.002*
**POSTOPERATIVE ADJUVANT TREATMENT**		
Yes	Reference	–
No	1.063 (0.606–1.862)	0.832
**TMT**		
(per 1-mm increase)	0.839 (0.731–0.964)	0.013*
**GNRI**		
(per 1-point increase)	0.983 (0.964–1.002)	0.077
**RECONSTRUCTIVE SURGERY**		
No	Reference	–
Yes	2.054 (1.141–3.698)	0.016*

Abbreviations: CI, confidence interval; GNRI, geriatric nutritional risk index; TMT, temporal muscle thickness. * Statistical significance ($p < 0.05$).

Table S3. Sensitivity analysis for overall survival additionally adjusted for primary tumor site.

Variable	Multivariate analysis	
	Hazard ratio (95% CI)	*p* value
**AGE**	1.042 (1.003–1.082)	0.034*
**SEX**		
Female	Reference	–
Male	0.815 (0.330–2.009)	0.656
**CLINICAL STAGE**		
I–II	Reference	–
III–IV	3.785 (1.279–11.203)	0.016*
**POSTOPERATIVE ADJUVANT TREATMENT**		
Yes	Reference	–
No	0.691 (0.343–1.391)	0.300
**TMT**		
(per 1-mm increase)	0.746 (0.622–0.894)	0.001*
**GNRI**		
(per 1-point increase)	0.974 (0.953–0.994)	0.013*
**PRIMARY TUMOR SITE**		
Oral cavity	Reference	–
Oropharynx	0.423 (0.156–1.150)	0.092
Hypopharynx	1.083 (0.538–2.176)	0.824
Larynx	0.766 (0.370–1.584)	0.472

Abbreviations: CI, confidence interval; GNRI, geriatric nutritional risk index; TMT, temporal muscle thickness. * Statistical significance ($p < 0.05$).

Table S4. Sensitivity analysis for overall survival additionally adjusted for reconstructive surgery.

**Variable**	**Multivariate analysis**	
	**Hazard ratio (95% CI)**	***p* value**
**AGE**	1.040 (1.001–1.080)	0.043*
**SEX**		
Female	Reference	–
Male	0.904 (0.347–2.357)	0.836
**CLINICAL STAGE**		
I–II	Reference	–
III–IV	4.554 (1.582–13.111)	0.005*
**POSTOPERATIVE ADJUVANT TREATMENT**		
Yes	Reference	–
No	0.783 (0.397–1.546)	0.482
**TMT**		
(per 1-mm increase)	0.744 (0.625–0.884)	0.001*
**GNRI**		
(per 1-point increase)	0.974 (0.954–0.995)	0.015*
**RECONSTRUCTIVE SURGERY**		
No	Reference	–
Yes	1.131 (0.647–1.978)	0.665

Abbreviations: CI, confidence interval; GNRI, geriatric nutritional risk index; TMT, temporal muscle thickness. * Statistical significance ($p < 0.05$).

Table S5. Sensitivity analysis using cohort-derived sex-specific median cutoffs for TMT.

Variable	DFS (HR, 95% CI)	*p* Value	OS (HR, 95% CI)	*p* Value
**Low TMT (sex-specific median)**	Reference		Reference	
**High TMT (sex-specific median)**	0.57 (0.36–0.92)	0.022	0.43 (0.23–0.78)	0.005
**GNRI (per 1-point increase)**	0.98 (0.96–1.00)	0.041	0.97 (0.95–0.99)	0.004
**Age (years)**	1.01 (0.98–1.03)	0.643	1.04 (1.01–1.08)	0.010
**Female sex**	Reference		Reference	
**Male sex**	0.69 (0.36–1.30)	0.249	0.81 (0.36–1.83)	0.609
**Clinical stage I–II**	Reference		Reference	
**Clinical stage III–IV**	3.35 (1.63–6.91)	0.001	4.33 (1.54–12.23)	0.006
**Postoperative adjuvant treatment: Yes**	Reference		Reference	
**Postoperative adjuvant treatment: No**	0.93 (0.56–1.54)	0.764	0.70 (0.38–1.28)	0.247

Sensitivity analyses were performed by redefining low TMT using cohort-derived sex-specific median values (men: 6.12 mm; women: 5.21 mm) to assess the robustness of the associations with survival outcomes. Cox proportional hazards models for DFS and OS were refitted using the same set of clinical covariates as in the primary analysis. Abbreviations: CI, confidence interval; DFS, disease-free survival; GNRI, geriatric nutritional risk index; HR, hazard ratio; OS, overall survival; TMT, temporal muscle thickness.

Table S6. Single-marker Cox model for overall survival with GNRI entered as a continuous variable.

Variable	HR	95% CI	*p* Value
**GNRI (per 1-point increase)**	0.97	0.95–0.99	0.004
**Age (years)**	1.05	1.02–1.09	0.001
**Sex (Male vs Female)**	0.76	0.33–1.72	0.509
**Clinical stage II (vs I)**	1.16	0.16–8.39	0.882
**Clinical stage III (vs I)**	3.17	0.70–14.41	0.135
**Clinical stage IV (vs I)**	7.51	1.76–32.17	0.007
**Postoperative adjuvant treatment (No vs Yes)**	0.83	0.45–1.52	0.532

Overall survival was examined using Cox proportional hazards models with GNRI entered as a continuous variable. Models were adjusted for age, sex, clinical stage, and postoperative treatment. Abbreviations: CI, confidence interval; GNRI, geriatric nutritional risk index; HR, hazard ratio.

Table S7. Pathology-adjusted complete-case sensitivity analyses for disease-free survival and overall survival.

Variable	DFS, HR (95% CI)	*p* Value	OS, HR (95% CI)	*p* Value
TMT, per 1-mm increase	0.86 (0.74–1.00)	0.044	0.76 (0.63–0.91)	0.003
GNRI, per 1-point increase	1.00 (0.97–1.02)	0.701	0.98 (0.96–1.00)	0.034
Age, per year	0.99 (0.96–1.02)	0.420	1.03 (0.99–1.07)	0.170
Male sex	1.02 (0.55–1.91)	0.941	0.93 (0.39–2.21)	0.866
Clinical stage III–IV vs. I–II	2.39 (1.07–5.34)	0.034	3.76 (1.15–12.27)	0.028
Postoperative adjuvant treatment, No vs. Yes	4.55 (2.08–10.00)	<0.001	1.59 (0.61–4.00)	0.341
Non-clear surgical margin vs. clear margin	1.03 (0.42–2.52)	0.952	0.76 (0.19–3.04)	0.703
Extranodal extension, present vs. absent	7.16 (3.78–13.58)	<0.001	2.92 (1.44–5.91)	0.003
Perineural invasion, present vs. absent	3.11 (1.90–5.10)	<0.001	2.14 (1.22–3.77)	0.008

Abbreviations: CI, confidence interval; DFS, disease-free survival; GNRI, geriatric nutritional risk index; HR, hazard ratio; OS, overall survival; TMT, temporal muscle thickness. Note: Complete-case sensitivity analyses were performed in 189 patients with complete data for surgical margin status, extranodal extension, and perineural invasion. Cox models were additionally adjusted for these pathological variables. Surgical margin status was coded as clear vs. non-clear, with close and positive margins classified as non-clear. Extranodal extension and perineural invasion were coded as absent vs. present.

Table S8. Model discrimination for overall survival assessed by optimism-corrected concordance index (C-index).

Outcome	Model	Variables	Apparent C-index	Mean optimism	Optimism-corrected C-index
OS	Model A	TMT + age + sex + clinical stage + postoperative treatment	0.7641	0.0700	**0.6941**
OS	Model B	GNRI + age + sex + clinical stage + postoperative treatment	0.7613	0.0563	**0.7050**
OS	Model C	TMT + GNRI + age + sex + clinical stage + postoperative treatment	0.7766	0.0678	**0.7087**

Internal validation was performed using bootstrap resampling (200 repetitions). Optimism-corrected C-index was calculated as apparent C-index minus mean optimism. While models including either TMT or GNRI demonstrated moderate discrimination, the combined model incorporating both TMT and GNRI achieved the highest optimism-corrected C-index, suggesting complementary prognostic value of these two parameters. Abbreviations: GNRI, geriatric nutritional risk index; OS, overall survival; TMT, temporal muscle thickness.

Table S9. Model comparison and incremental value of adding TMT and GNRI for DFS and OS.

(A) Disease-free survival (DFS)

Model	Predictors	df	Log likelihood	AIC	BIC	Harrell's C (apparent)
A_dfs	TMT + covariates	7	−382.5545	779.1089	802.6707	0.7219
B_dfs	GNRI + covariates	7	−383.5523	781.1046	804.6664	0.7245
C_dfs	TMT + GNRI + covariates	8	−381.2581	778.5162	805.4440	0.7277

Incremental value of Model C (nested model comparison)

Comparison	LR $\chi^2(1)$	p value	ΔAIC	ΔBIC	ΔC (bootstrap, 1,000 reps)	95% CI	p value
C_dfs vs A_dfs (add GNRI)	2.59	0.1074	−0.5927	+2.7733	0.0127748	−0.00895 to 0.03450	0.249
C_dfs vs B_dfs (add TMT)	4.59	0.0322	−2.5884	+0.7776	0.0205724	−0.00494 to 0.04608	0.114

(ΔAIC = AIC_C − AIC_base, ΔBIC = BIC_C − BIC_base)

(B) Overall survival (OS)

Model	Predictors	df	Log likelihood	AIC	BIC	Harrell's C (apparent)
A_os	TMT + covariates	7	−264.4608	542.9216	566.4835	0.7641
B_os	GNRI + covariates	7	−265.9699	545.9397	569.5016	0.7613
C_os	TMT + GNRI + covariates	8	−262.0237	540.0475	566.9753	0.7766

Incremental value of Model C (nested model comparison)

Comparison	LR $\chi^2(1)$	p value	ΔAIC	ΔBIC	ΔC (bootstrap, 1,000 reps)	95% CI	p value
C_os vs A_os (add GNRI)	4.87	0.0273	−2.8741	+0.4918	0.0045496	−0.02985 to 0.03894	0.795
C_os vs B_os (add TMT)	7.89	0.0050	−5.8922	−2.5263	0.0307823	−0.00291 to 0.06447	0.073

Abbreviations: AIC, Akaike Information Criterion; BIC, Bayesian Information Criterion; CI, confidence interval; GNRI, geriatric nutritional risk index; TMT, temporal muscle thickness. **Common covariates in all models:** age, sex, clinical stage (cstage), and postoperative treatment (post_tx). **Model A:** TMT +

covariates **Model B:** GNRI + covariates **Model C:** TMT + GNRI + covariates Harrell's C-index values are **apparent** (from estat concordance). ΔC was assessed by bootstrap resampling (1,000 replications) as the difference in Harrell's C between the compared models.
